# Supplementary material for: Effect of Angiotensin II on Bone Erosion and Systemic Bone Loss in Mice with Tumor Necrosis Factor-Mediated Arthritis
Source: Int J Mol Sci. 2020 Jun 10;21(11):4145. doi: 10.3390/ijms21114145 (PMC7312645; doi:10.3390/ijms21114145)
Supplement: Supplementary file 1 [file ijms-21-04145-s001.pdf]

Supplementary Methods

Western Blotting

Protein expression in the tissues was determined by Western blot analysis, as described previously [1]. After anesthetization, fore paws were isolated from 16-week-old wild-type and TNFtg mice. The tissues were immediately freezed by liquid nitrogen, crushed by using a tissue pulverizer (IKEDA trading Corporation, Tokyo, Japan), and soaked in the RIPA lysis buffer (Sigma-Aldrich) containing a protease inhibitor cocktail (P8340, Sigma-Aldrich). After centrifugation, supernatants were collected, and protein concentrations were determined using a BCA protein assay kit (Thermo Fisher Scientific). Protein samples (10 μg/lane) were resolved by sodium dodecyl sulfate-polyacrylamide gel electrophoresis and transferred to nitrocellulose membranes. After blocking, the membranes were incubated with the indicated primary antibodies, followed by incubation with the appropriate horseradish peroxidase-conjugated secondary antibodies. The band was detected using SuperSignal West chemiluminescent substrate (Thermo Fisher Scientific) and visualized using LAS-4000 (GE Healthcare, Little Chalfont, UK). The antibodies used in this study were as follows: anti-AT1R (ab47162; Abcam, Cambridge, UK) and anti-Actin (A2066; Sigma-Aldrich).

Immunohistochemistry

Immunohistochemistry analyses were performed on paraffin-embedded ankle joint tissue specimens obtained from 16-week-old wild-type and TNFtg mice. The slides were processed for staining using an automated immunostainer (Ventana XT System Discovery; Ventana Medical Systems, Tucson, AZ, USA). Epitope retrieval was accomplished with CC1 solution (Ventana Medical Systems). Anti-AT1R antibody (Ab47162, Abcam, diluted to 10 μg/mL) was incubated at 37 °C for 32 minutes. Tissue specimens processed without the primary antibody were used as negative controls.

Osteoclast Differentiation Assay

Primary mouse bone marrow cells were isolated from the long bones of 10-week-old wild-type mice. Bone marrow cells were cultured on Petri dishes for 2–4 h at 37 °C in 5% CO_2. Non-adherent bone marrow cells were collected and re-seeded on 48-well plates at a density of 5.0×10^4 cells/well and incubated for 2 days in α-MEM supplemented with 10% FBS containing macrophage colony-stimulating factor (M-CSF; 25 ng/mL, PeproTech, Rocky Hill, NJ, USA) at 37 °C in 5% CO_2. After pre-culture for 2 days, yielded bone marrow-derived macrophages were stimulated with RANKL (50 ng/mL, PeproTech) and Ang II at the indicated concentrations in the presence of M-CSF (25 ng/mL) for an additional 3 days. The formation of tartrate-resistant acid phosphatase (TRAP)-positive multinucleated cells (TRAP + MNCs) was visualized by TRAP staining (Sigma-Aldrich). TRAP + MNCs with three or more nuclei were counted as osteoclasts.

Osteoblasts were isolated from the newborn mouse calvaria as described previously [2]. Co-cultures with bone marrow cells were performed in the presence of 10 nM 1α,25-dihydroxyvitamin D3 (Cayman Chemical Company, Ann Arbor, MI, USA) and 1 μM prostaglandin E2 (Cayman Chemical Company) for 7 days. The cells were stimulated with Ang II (0.01–10μM) for 7 days. After TRAP staining, TRAP + MNCs were counted under light microscopy.

References

1. Nagasu, A.; Mukai, T.; Iseki, M.; Kawahara, K.; Tsuji, S.; Nagasu, H.; Ueki, Y.; Ishihara, K.; Kashihara, N.; Morita, Y. Sh3bp2 Gain-Of-Function Mutation Ameliorates Lupus Phenotypes in B6.MRL-Fas(lpr) Mice. *Cells* **2019**, *8*, doi:10.3390/cells8050402.
2. Fujita, S.; Mukai, T.; Mito, T.; Kodama, S.; Nagasu, A.; Kittaka, M.; Sone, T.; Ueki, Y.; Morita, Y. Pharmacological inhibition of tankyrase induces bone loss in mice by increasing osteoclastogenesis. *Bone* **2018**, *106*, 156–166, doi:10.1016/j.bone.2017.10.017.
